# Biomechanical factors and atherosclerosis localization: insights and clinical applications

**DOI:** 10.3389/fcvm.2024.1392702

**Published:** 2024-07-25

**Authors:** Elena Bacigalupi, Jacopo Pizzicannella, Gianluca Rigatelli, Luca Scorpiglione, Melissa Foglietta, Greta Rende, Cesare Mantini, Franco M. Fiore, Francesco Pelliccia, Marco Zimarino

**Affiliations:** ^1^ Department of Neuroscience, Imaging and Clinical Sciences, “G. D'Annunzio” University of Chieti-Pescara, Chieti, Italy; ^2^ Department of Engineering and Geology, University “G. d’ Annunzio” Chieti-Pescara, Pescara, Italy; ^3^ Cardiology Department, Ospedali Riuniti Padova Sud, Monselice, Italy; ^4^ Cardiology Department, SS. Annunziata Hospital, Chieti, Italy; ^5^ Division of Vascular Surgery, SS. Annunziata Hospital, Chieti, Italy; ^6^ Department of Cardiovascular Sciences, University Sapienza, Rome, Italy

**Keywords:** biomechanical factors, wall shear stress, axial plaque stress, plaque structural stress, computational fluid dynamic

## Abstract

Although the entire vascular bed is constantly exposed to the same risk factors, atherosclerosis manifests a distinct intra-individual pattern in localization and progression within the arterial vascular bed. Despite shared risk factors, the development of atherosclerotic plaques is influenced by physical principles, anatomic variations, metabolic functions, and genetic pathways. Biomechanical factors, particularly wall shear stress (WSS), play a crucial role in atherosclerosis and both low and high WSS are associated with plaque progression and heightened vulnerability. Low and oscillatory WSS contribute to plaque growth and arterial remodeling, while high WSS promotes vulnerable changes in obstructive coronary plaques. Axial plaque stress and plaque structural stress are proposed as biomechanical indicators of plaque vulnerability, representing hemodynamic stress on stenotic lesions and localized stress within growing plaques, respectively. Advancements in imaging and computational fluid dynamics techniques enable a comprehensive analysis of morphological and hemodynamic properties of atherosclerotic lesions and their role in plaque localization, evolution, and vulnerability. Understanding the impact of mechanical forces on blood vessels holds the potential for developing shear-regulated drugs, improving diagnostics, and informing clinical decision-making in coronary atherosclerosis management. Additionally, Computation Fluid Dynamic (CFD) finds clinical applications in comprehending stent-vessel dynamics, complexities of coronary bifurcations, and guiding assessments of coronary lesion severity. This review underscores the clinical significance of an integrated approach, concentrating on systemic, hemodynamic, and biomechanical factors in atherosclerosis and plaque vulnerability among patients with coronary artery disease.

## Key points

• Biomechanical factors are key players in coronary atherosclerosis and can be assessed *in vivo* through computational fluid dynamics techniques.
• Biomechanical factors contribute to valuable prognostic information beyond anatomical and physiological plaque characteristics in the localization and evolution of coronary atherosclerosis.
• Computational fluid dynamics offers clinical applications in the assessment of plaque vulnerability, coronary lesion severity, stent-vessel interactions, and coronary bifurcation complexities.

## 1 Introduction

Atherosclerosis, a chronic systemic disease marked by inflammation and fibro-proliferation, predominantly affects the intima of large and medium-sized elastic and muscular arteries (1). It is the leading cause of morbidity and mortality worldwide and presents its main clinical manifestation as ischemia, which can damage the heart, brain, or lower extremities. Despite uniform exposure to the same risk factors across the vascular bed, the formation and advancement of atheromatous lesions follow a unique pattern, frequently occurring in certain segments of the arterial system. This peculiar distribution might stem from a variable responsiveness to risk factors or differences in histopathology and blood flow, and has relevant clinical implications, as the prognosis of the disease varies according to localization (2).

The role of risk factors in atherosclerosis is complex, encompassing anatomical, physiological, and behavioral aspects. Conventional risk factors such as hypertension, hypercholesterolemia, tobacco smoking, diabetes mellitus, age, family history, and obesity, are well established (3). Recent evidence also highlight the significant role of inflammation and the immune system as key mechanism in the pathophysiology of cardiovascular disease (4).

In the context of atherosclerosis, biomechanical forces such as Wall Shear Stress (WSS), Axial Plaque Stress (APS), and Plaque Structural Stress (PSS) are pivotal in its localization and progression (5, 6). These forces, arising from blood flow dynamics, interact with the endothelial lining of blood vessels, influencing the formation, growth, and vulnerability of atherosclerotic plaques. The integration of advanced computational fluid dynamics (CFD) with imaging techniques like Coronary Computed Tomography (CT) and Intravascular Ultrasound (IVUS) has enabled a more detailed analysis of these biomechanical factors, providing new insights into their role in the localization and progression of coronary atherosclerosis.

This review explores the growing importance of biomechanical factors, focusing on recent discoveries related to WSS, APS, and PSS, along with the evolving role of CFD in the diagnosis and treatment of atherosclerosis.

## 2 Biomechanical factors: physiophatologic role and current evidence

**Figure 1:**
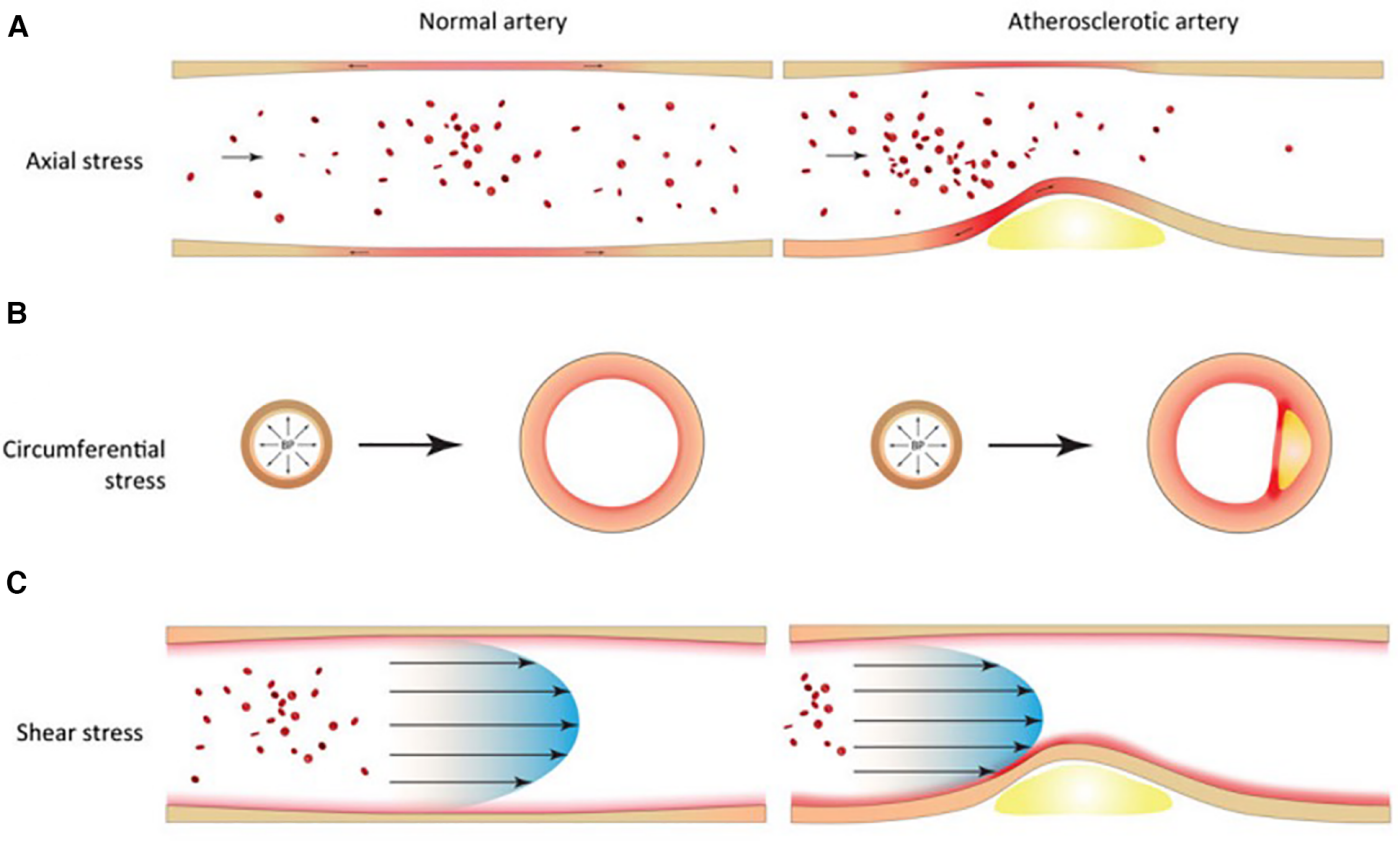
Biomechanical factors in normal and atherosclerotic vascular tissues. (**A**) Axial stress arises from longitudinal stretching of tethered blood vessels. (**B**) Circumferential stress arises from radial expansion and recoil over the cardiac cycle. (**C**) Shear stress arises from the frictional force of blood flowing against the vessel wall.

The oscillatory nature of blood flow exerts mechanical stress on vascular tissues, significantly influencing vessel biology. In particular, blood flow generates circumferential, axial and shear stress in coronary vessels (Figure 1). Extensive research over the years has heightened our understanding of these forces and their critical role in the development and pathology of atherosclerotic plaques. Marked oscillations of blood pressure - as measured by blood pressure variability - are associated with both depressed left ventricular systolic function and target organ damage (7). Innovations in medical imaging and computational approaches have enabled more accurate *in vivo* assessments of these biomechanical stress, offering key insights into plaque localization and progression (8).

To analyze biomechanical factors in human coronary arteries, a combination of imaging techniques and CFD is needed. Imaging is essential for capturing the three-dimensional structure of the coronary artery. The geometrical data is then given to a CFD software program that compute the velocity and shear stress distribution. The most precise method involves using biplane ANGiography combined with intravascular UltraSound (ANGUS) (9). Alternatively, CT or MRI angiography can approximate the 3D orientation of IVUS images for those cases where biplane angiography is not feasible. In addition to structural information, CFD analysis necessitates further inputs, notably the flow rate through the coronary artery. This can be gauged through either invasive techniques, such as a coronary pressure/flow wire, or non-invasive methods, like monitoring blood pressure.

While CFD simulations compute WSS and APS based on the velocity of blood flow, calculating PSS involves a deeper understanding of the mechanical characteristics of plaque components. This includes the magnitude of forces applied to the plaque and the degree of resultant plaque deformation. To compute PSS, an engineering approach known as finite element analysis (FEA) is employed. FEA considers the dynamic forces acting on the plaque and reconstructs 2D/3D images using IVUS, VH-IVUS, or OCT, thereby estimating PSS and its fluctuation throughout the cardiac cycle. Alternatively, PSS can be calculated using fluid-structure interaction (FSI) simulations. This method aims to integrate both the cyclic fluid dynamics and structural mechanical forces to achieve a comprehensive solution (10). While FSI enables the quantification of both WSS and PSS within a single artery, the engineering processes involved are highly complex, which limits its current use in clinical settings.

### 2.1 Wall shear stress

**Figure 2:**
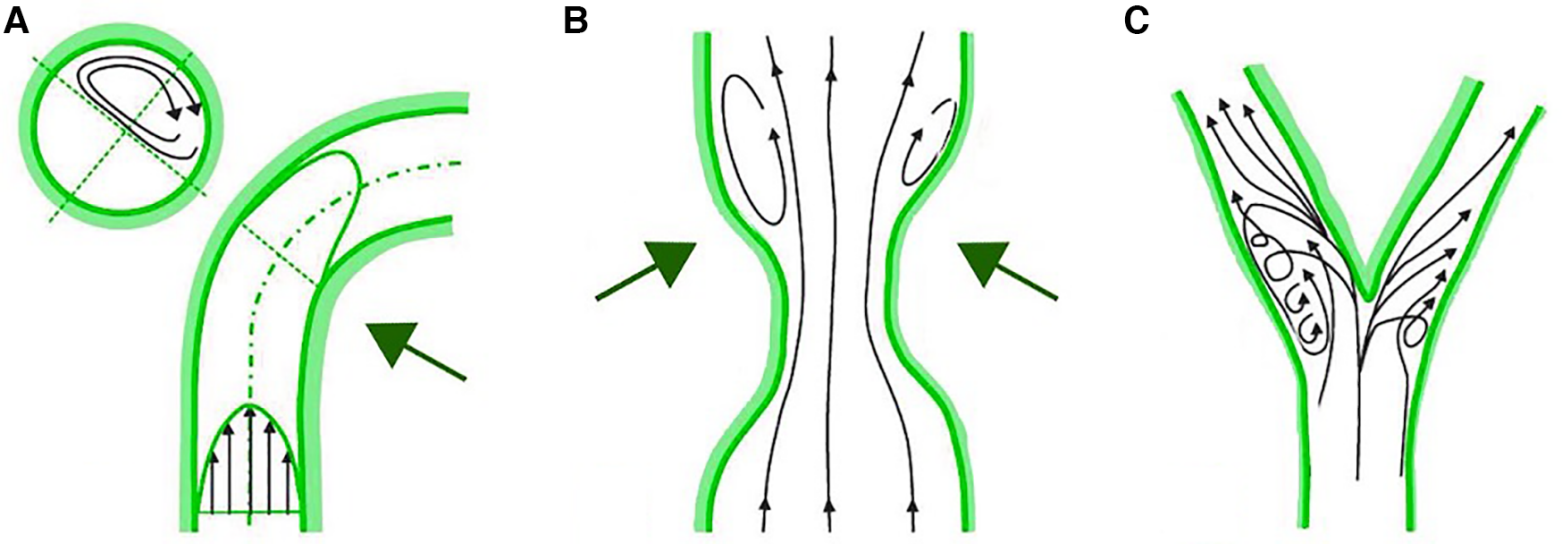
Complex flow patterns in coronary arteries. (**A**) Inner curvature of vessels. (**B**) Downstream part of stenosed segments. (**C**) Lateral wall of bifurcations.

WSS is the parallel frictional force exerted by blood flow on the endothelium of the arterial wall. It is expressed in units of force/unit area [N/m^2^ or Pascal (Pa) or dyne/cm^2^; 1 N/m^2 ^= 1 Pa = 10 dyne/cm^2^]. In straight arterial segments, WSS is pulsatile and unidirectional, fluctuating between 15 and 70 dyne/cm^2^ over the cardiac cycle. The mechanotransduction of WSS via endothelial cell transmembrane proteins influences intracellular enzyme functions, gene expression, synthesis of proteins and micro-RNAs. These processes modulate endothelial cell structure and function, influencing the surrounding environment, and the balance between inhibition and promotion of atherosclerosis. WSS above 15 dyne/cm^2^ promotes a quiescent, antiproliferative, antioxidant, and antithrombotic phenotype, alongside an atheroprotective gene expression profile. In critical geometrically irregular points of the coronary tree, complex flow dynamic patterns occur, with swirling flow, flow separation, and flow reversal that generates low and/or oscillatory WSS. These regions are the inner curvature of vessels, hips, or the lateral wall of bifurcations and the downstream part of stenosed segments (Figure 2). Low and oscillatory WSS (<10 dyne/cm^2^) results in inflammation and proatherogenic pathways of the endothelium. Low WSS decreases the production of fibrinolytics, vasodilators, and antioxidants and increases the expression of growth factors, oxidative elements, vasoconstrictors, acute inflammatory mediators and proteolytic enzymes. This sequence of endothelial dysfunction and acute inflammation can perpetuate injury that contributes to plaque growth and arterial remodeling. Notably, areas subjected to low WSS experience accelerated plaque progression, even in regions initially free of plaque (11).

The early stages of atherosclerosis feature positive remodeling of coronary vessels, which temporarily prevents narrowing due to plaque protrusion. However, segments exposed to low WSS remain susceptible to further atherogenesis, leading to a detrimental cycle of ongoing endothelial damage, plaque expansion, and deteriorating flow dynamics (12). This phenomenon continues until the plaque occupies a significant part of the vessel's area (around 40%), at which point luminal compensation fails. As a result, the plaques that protrudes into the lumen are exposed to higher WSS. This alteration in WSS magnitude and distribution creates a biomechanical environment that not only favors downstream plaque progression but also changes plaque vulnerability. Whereas higher WSS is typically considered atheroprotective in healthy vessels, its presence in arteries with protruding plaques can promote further vulnerable changes leading to thin-cap fibroatheroma (TCFA) morphology. This phenomenon involves an upregulation in nitric oxide production and the activation of metalloproteinases, which contribute to the regression of fibrous caps, thus destabilizing the plaques (13, 14). Even endothelial progenitor cells have been hypothesized to act as mediators in the link between traditional risk factors, WSS, and atherosclerosis, but evidence has been controversial (15).

Numerous studies support these pathophysiological evaluations, elucidating the role of WSS in the origin and progression of coronary atherosclerosis. The PREDICTION study, an IVUS-based CFD analysis, identified low WSS as a key factor in increasing plaque burden and reducing lumen area (16). Low WSS was also predictive of lesions requiring percutaneous intervention in a majority of asymptomatic chronic coronary syndrome (CCS) patients during the follow-up. Similarly, Bourantas and colleagues demonstrated that low WSS in non-culprit vessels of patients with myocardial infarction (MI) was an independent indicator of disease progression (17). Evidence underscores also the role of WSS in changing plaque vulnerability, leading to destabilization and rupture (18). In the EMERALD study, coronary computed tomographic angiography (CTA), documented that, in patients with acute coronary syndrome (ACS), culprit lesions were more likely to exhibit both adverse plaque characteristics (APC) - positive remodeling, low attenuation, and varied calcifications - and adverse hemodynamic characteristics (AHC), namely higher proximal WSS. Lesions exhibiting both APC and AHC were associated with a higher risk of subsequent acute events, compared with patient without APC/AHC and those with either APC or AHC (19). The prognostic role of WSS was also demonstrated in 441 patients with chronic coronary syndrome with significant lesions enrolled in the medical therapy arm of the FAME II trial (20). The authors found that higher WSS in the proximal segments of coronary lesions correlates with MI within 3 years. Similarly, Zuin et al. demonstrated that in angiographically non-significant left-main (LM) bifurcation disease, both higher proximal WSS of each branch and higher WSS of the entire lesion predicted the occurrence of bifurcation-located MI over the following 3 years (21). Parallel findings by Fukumoto et al. using IVUS (22) and Jin (23) in an OCT-based CFD study reinforced the link between higher WSS and plaque rupture locations.

### 2.2 Axial plaque stress

Axial stress comes from the longitudinal stretching of tethered blood vessels and represents the fluid stress aligned along the vessel's central axis throughout the cardiac cycle. Non-diseased arteries maintain minimal levels of axial stress. In atherosclerotic arteries with flow obstructions, the imbalance of external hemodynamic forces across lesions increases the axial stress and overall plaque strain. This axial stress is of a higher magnitude than WSS and may become concentrated at the upstream and downstream segments of a plaque. The increase of axial stress due to flow obstruction and subsequent pressure gradients may play a role in plaque rupture (24).

Choi and colleagues evaluated APS acting on stenotic lesions and its relationship with lesions' length and geometry (25). The authors divided lesions in upstream- and downstream-dominant according to the localization (upstream or downstream, respectively) of steeper radius change, where radius change refers to the difference between the lesion starting (or ending) point radius and the radius at the location of minimum lumen area. In addition, a negative correlation between APS and lesion length was found and APS was higher in the upstream segment of upstream-dominant lesions and in the downstream segment of downstream-dominant lesions. This offers insight into why short, focal lesions are more prone to rupture than long ones and elucidates the paradoxical phenomenon of downstream rupture. Furthermore, the EMERALD study found that axial stress had an incremental value for predicting ACS, resulting in higher APS in culprit compared to non-culprit lesions.

### 2.3 Plaque structural stress

Circumferential stress is generated through the radial dilation and contraction of arteries within the cardiac cycle. In healthy arteries, this force is uniformly distributed along the artery wall due to homogenous tissue composition. Plaque Structural Stress (PSS) is the circumferential stress located inside an atherosclerotic plaque as a consequence of vessel expansion induced by arterial pressure. The magnitude of PSS is influenced by factors such as plaque composition, architecture, and lumen geometry. In particular, PSS increases with increasing lumen area, eccentricity, and necrotic core, and decreases in the presence of dense calcium (26). In addition, there is a reciprocal relationship between plaque composition and morphology, the thickness of fibrous cap (27), WSS, and PSS itself. Recently (28), both in areas of plaque progression and regression, higher PSS was associated with larger increases in necrotic core, leading to vulnerable phenotype. Indeed, PSS was higher in patients presenting with ACS vs. CCS (29), in peri-minimum lumen area (MLA) of plaques showing rupture vs. no rupture (30), and in non-culprit lesions leading to MACEs vs. no MACEs even with similar characteristics (31).

### 2.4 Radial wall strain

Radial wall strain (RWS) similarly reflects the interplay between cyclic pulsatile intravascular pressure and vessel wall tissue composition, resulting in higher values for vulnerable plaque components like lipids or macrophages, and lower values for fibrous tissue or calcium. Coronary strain can be measured using computational dynamic techniques with FEA from IVUS or OCT images. However, this requires intracoronary imaging devices and complex analytical processes. Recently, a novel artificial intelligence (AI) method was developed to calculate RWS from a single angiographic projection (RWS-Angio) and has been validated against corresponding intravascular images (32). This method provides a simplified and cost-effective tool for assessing plaque biomechanical characteristics, potentially accessible in all cath labs. Studies have demonstrated a strong correlation between RWS-Angio and intracoronary imaging features of plaque vulnerability in intermediate coronary stenosis (33). Additionally, RWS-Angio has shown increased and independent prognostic value in predicting target vessel failure (TVF), regardless of FFR values (34). Given the recent focus on the unfavorable clinical outcomes of non-flow limiting coronary plaques with high-risk characteristics (35) and the ongoing debate about the revascularization of such lesions (36), the ability to estimate RWS from a routine diagnostic coronary angiogram could provide significant prognostic value in managing intermediate coronary lesions and improving CAD treatment strategies.

## 3 Computational fluido dynamics models and clinical applications

### 3.1 Assessment of plaque vulnerability and rupture

The primary clinical outcome of atherosclerosis is ischemia, leading to significant damage in the heart, brain, or lower limbs. However, it is noteworthy that such lesions tend to manifest predominantly in specific regions of the arterial tree (37). CFD has been recently applied to the pathophysiology of atherosclerosis to understand the mechanisms underlying plaque progression. Over the years, both invasive and non-invasive imaging studies have consistently demonstrated that evaluating anatomical characteristics of plaque provides a more accurate prediction of cardiac event risks than merely assessing the extent of lumen obstruction (38, 39). Nevertheless, the evaluation of plaque vulnerability based solely on imaging features has its limitations. As the PROSPECT study showed (40), only a small proportion (<10%) of thin-cap fibroatheroma plaques progress to cause clinical events over 3 years; similarly, Motoyama et al. (41) documented that the majority (84%) of coronary high-risk plaques defined by CTA failed to cause any event. While these morphological characteristics are crucial indicators of vulnerable plaques, there is a need for additional markers to identify plaques that are likely to advance to clinical significance. As mentioned before, WSS, APS, PSS and RWS account for changes in plaque composition, leading to plaque vulnerability and rupture. Recent investigations have also delved into the phenomenon of cavitation, which physically occurs when fluid pressure in a particular area falls below the vapor pressure (42). CFD studies demonstrated that cavitation, triggered by both concentric and eccentric coronary artery stenosis, travels downstream, forming microbubbles that burst when fluid pressure dips below the vapor pressure in a local thermodynamic condition. This might damage endothelial surfaces, promoting thrombosis (43).

None of these biomechanical and anatomical factors alone can however explain the complex phenomenon of plaque rupture and acute thrombosis. They act as concurrent factors in the precipitation of atherosclerotic disease. Future prospective studies are warranted to understand whether the assessment of biomechanical factors acting on a coronary lesion can provide predictive value beyond anatomical and functional data and to explore its potential clinical application in guiding preventive and therapeutic measures.

### 3.2 Assessment of stent-vessel interaction

CFD is increasingly applied to assess how stent architecture influences coronary blood flow dynamics. Recent studies suggest that the geometry created by an implanted stent can alter local blood flow and wall shear stress (WSS) distribution. This alteration can predispose certain vessel wall areas to neointimal hyperplasia and restenosis (44), rather than promoting the typical shear stress-induced re-endothelialization (45). However, in such cases, the precise mechanism driving the excessive neointimal proliferation is still unclear.

When a stent, which is comparatively rigid, is implanted in a curved vessel like a coronary artery, it alters the segment's shape, essentially straightening it. This results in increased curvature at the stent's edges, creating zones of low WSS and potential flow reversal. Furthermore, the stent deployment - considering the applied pressure, the design of the stent, and strut dimensions - may result in the struts extending into the lumen, which modifies velocity and the distribution of shear stress, thereby creating zones of high and low WSS. Although *in vitro* studies have correlated these flow disturbances to increased platelet adhesion, there is a lack of studies evaluating clinical restenosis outcomes. Furthermore, post-stent implantation, if the diameter of the restored lumen within the stent surpasses that of the artery's proximal section, a sudden lumen expansion just downstream of the stent's leading edge occurs. Such flow patterns are comparable with low-WSS flow recirculation documented downstream stenosis. LaDisa et al. (46, 47) demonstrated that low WSS occurs predominantly in the proximal and distal edges of the stent. Such low WSS regions are also notable behind struts, especially those protruding into the lumen at steep angles to the flow direction (48), and were associated with neo-atherosclerosis (49). Moreover, *in vitro* experiments exploring the impact of stent geometry on platelet adhesion and aggregation, found that localized platelet accumulation is influenced by flow convection, implying that vascular response to stent deployment can be somewhat governed by the hemodynamics associated with stent configuration (50).

CFD models show promise in understanding how stents interact with blood vessels. Optimizing stent design must account for the local flow patterns induced by the stent's deployment, to avoid the fluid dynamic conditions that can promote neointimal hyperplasia and subsequent restenosis.

### 3.3 Assessment of bifurcations

**Figure 3:**
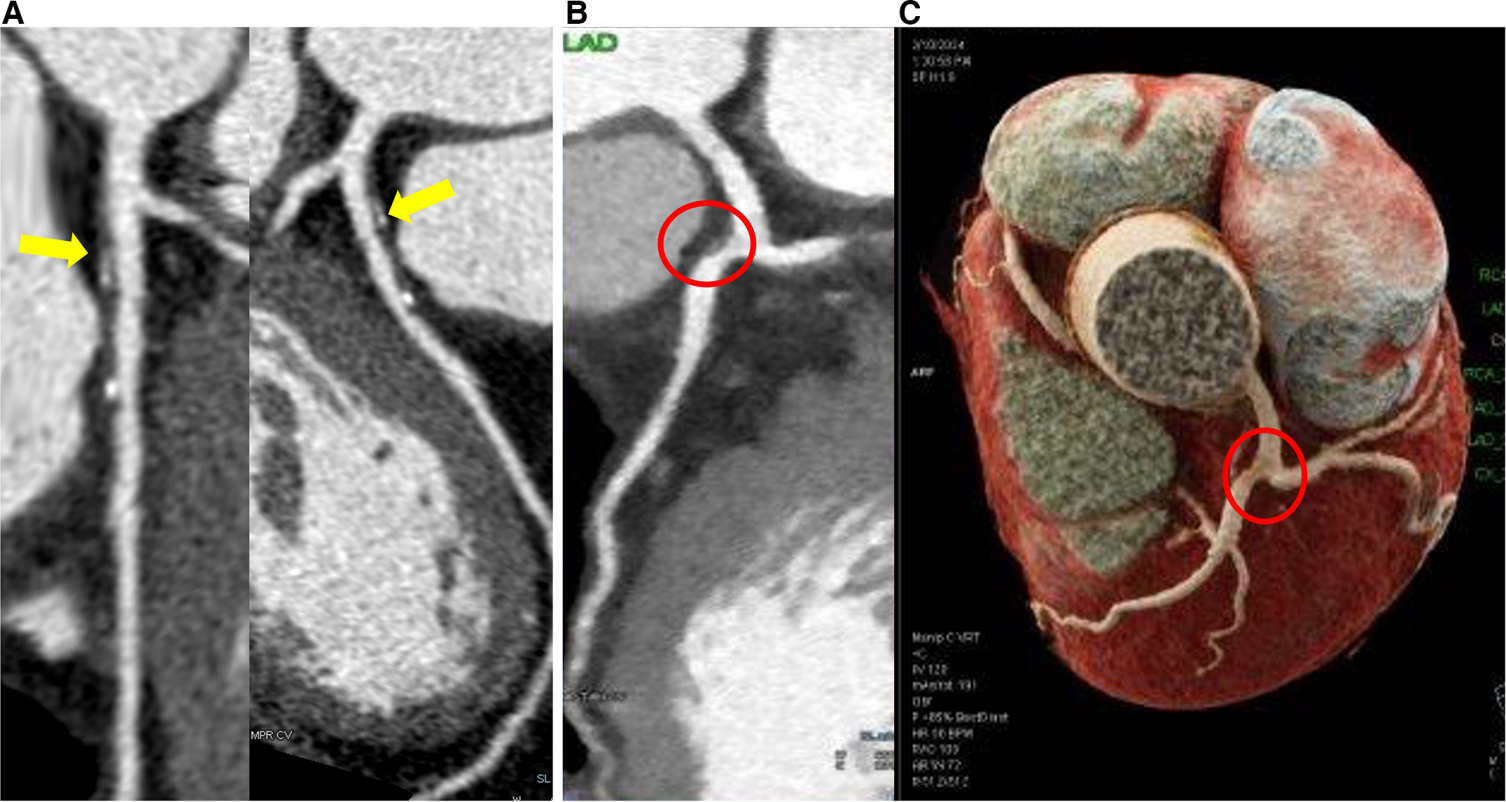
Coronary computed tomography angiography shows plaques located at the lateral wall of the bifurcation. (**A**) Eccentric partially calcified plaque in the left anterior descending coronary artery immediately downstream of the left main bifurcation. (**B**) Eccentric vulnerable plaque in lateral wall of left anterior descending coronary artery immediately downstream of the left main bifurcation (red circle) in 2D and 3D reconstruction (**C**).

In bifurcations, the flow divides into two daughter branches and changes in direction. Consequently, the faster moving particles will impinge on the inner wall areas of the side branch (SB) proximal to the carina, while in the hips or lateral wall occurs low and oscillatory WSS with flow separation (Figure 3). This complex flow pattern is influenced by several factors, including the bifurcation angle, the size of the SB, and how the blood flow is distributed between the branches.

Coronary bifurcation revascularization poses challenges for interventionalists. Factors such as the dimensions of the SB, the extent of lesions, calcification levels, the morphology of the bifurcation carina, and the angles formed between the two branches demand careful consideration in treatment planning. Bifurcation stenting inevitably affects coronary flow patterns and induces geometric changes in both the main (MB) and SB. CFD was used to anticipate the results of complex bifurcation stenting and to understand how bifurcation rheology changes following stenting with various devices and techniques. Key hemodynamic parameters assessed at stented coronary bifurcations through CFD include WSS, time-averaged wall shear stress (TAWSS), oscillatory shear index (OSI), and relative residence time (RRT). TAWSS quantifies the mean WSS over a cardiac cycle, OSI reflects the variability of WSS, and RRT combines TAWSS and OSI to indicate the duration that blood particles remain near the vessel wall. Areas of low and oscillatory WSS with high OSI and RRT, such as the lateral wall of stented MB, are prone to intra-stent restenosis (ISR). Factors like the number and thickness of stent struts (51), the bifurcation angle, and any stent malapposition significantly affect the development of adverse hemodynamic conditions and can promote ISR and thrombus formation.

Thus, choosing the optimal stenting strategy, whether a provisional single-stent or a more complex two-stent approach, is of paramount importance. Single-stent technique tends to leave vulnerable areas opposite the flow divider (52), while dual-stent techniques can create secondary flow disturbances and low WSS due to strut apposition at the carina, strut layering and protrusion into the MB, which are associated with ISR or thrombosis. The best double stenting strategies with the most favorable fluid dynamics are under debate (53–55) and efficiency of single or double stenting depends also on bifurcation anatomical complexity. A recent OCT derived-CFD study evaluating RRT in complex left main bifurcation disease suggested for example that RRT is increased after cross-over stenting while is substantially unchanged after double stenting techniques (56). Anyway, when approaching a bifurcation stenting procedure, careful planning is crucial, as the deployment of unplanned bailout additional stents is associated with adverse events (57).

The application of CFD in this context may represent a powerful tool to guide both PCI strategies and the type and duration of antithrombotic therapy after bifurcation PCI (58) in order to predict adverse events and identify the optimal strategy to improve clinical outcomes.

### 3.4 Assessment of the functional severity of coronary lesion

In recent years, the practice of coronary revascularization has been revolutionized by physiology-guided approaches, leading to improved outcomes in the management of CAD, compared to revascularization based solely on angiographic data. While several non-hyperemic pressure ratios have been developed to expand the use of physiology beyond FFR and its adenosine-induced hyperemia issues, the integration of these methods into routine practice remains limited, due to prolonged procedural durations and risks associated with guidewire use (59, 60). Addressing these challenges, computational approaches have emerged with Quantitative Flow Ratio (QFR) as the most validated technique (61), based on a three-dimensional vessel reconstruction and estimation of its contrast media flow velocity. QFR employs three-dimensional quantitative coronary angiography (3D-QCA) in combination with CFD to calculate the functional impact of coronary stenosis. This angiography-based method offers an accurate alternative to estimate FFR, bypassing the need for coronary wiring or the induction of hyperemia. Strong agreement between QFR and traditional FFR measurements have been observed (62, 63), including post-PCI assessment (64, 65) and prognostication in patients with left main or multi-vessels disease (66). Furthermore, QFR technology can predict the post-PCI functional outcomes through virtual angioplasty. This “virtual angioplasty” could be pivotal in strategizing revascularization plans and potentially forecasting procedural outcomes. Recent investigations exploring the relationship between QFR-obtained “virtual angioplasty” and wire-based post-PCI functional evaluations gave conflicting results, especially in tandem and complex lesions (67–69).

## 4 Conclusion and future prospective

Biomechanical factors are fundamental in coronary atherosclerosis formation and progression and should be considered beyond anatomical and physiological plaque characteristics. The application of biomechanical models in patient studies is proving to be a game-changer, significantly deepening our understanding of how shear stress interacts with endothelial function and the pathogenesis of atherosclerosis. Moreover, CFD applications extend beyond plaque assessment, evaluating stent-vessel interactions, coronary bifurcation complexities, and coronary lesion severity.

The advancements in noninvasive imaging and the understanding of blood flow properties through CFD models will set the basis for a personalized approach. This will enable tailored, patient-specific, or even plaque-specific solutions to various issues. The assessment of plaque vulnerability and the risk of rupture, along with the consequent need for revascularization, through the evaluation of biomechanical factors beyond anatomical and physiological characteristics will be explored. Determining the optimal stent design that minimizes damage to the coronary wall becomes a feasible consideration. While numerous issues still require resolution before incorporating these techniques into clinical practice, the integration of noninvasive imaging and biomechanical modeling will support cardiologists in improving diagnosis and establishing optimal treatment strategies for cardiovascular disease.
